# Psoriatic flare following Oxford-AstraZeneca ChAdOx1 COVID-19 and influenza vaccines

**DOI:** 10.34172/hpp.2021.47

**Published:** 2021-12-19

**Authors:** Natalie Teh, Liang Joo Leow

**Affiliations:** ^1^Faculty of Medicine, University of New South Wales, Sydney, Australia; ^2^Aesthetic Dermatology, Sydney, Australia

## Dear Editor,

Psoriatic flares following Pfizer-BioNTech, CoronaVac (Sinovac Biotech), Moderna, and Oxford-AstraZeneca COVID-19 vaccines have recently been reported.^1-3^ We present the first case of treated moderate facial and palmoplantar psoriasis, where a flare of moderate plaque psoriasis of the body resulted from the Oxford-AstraZeneca vaccine (AZ).

A Caucasian man of 80 years on biologic therapy with tildrakizumab experienced three psoriatic flares in close succession after AZ dose 2 and an intervening influenza vaccination (MF59®-adjuvanted quadrivalent influenza vaccine, Seqirus) between AZ doses.

His history of psoriasis involved chronic facial and palmoplantar plaques that gradually worsened over 30 years despite oral methotrexate and acitretin, resulting in moderate facial, and severe palmoplantar, erythema with 30% and 100% areas of involvement, respectively; warranting biologic therapy.

AZ dose 1 was administered uneventfully, followed by influenza vaccination 21 days after. Nine days after influenza vaccination, the first psoriatic flare occurred, with trunk, head, and limb involvement (PASI 11.4). This responded to oral prednisolone (50 mg to 12.5 mg daily over nine days) and betamethasone dipropionate 0.05% ointment. A second flare occurred 26 days after the first, which also responded to the same corticosteroid regimen.

**Figure 1:**
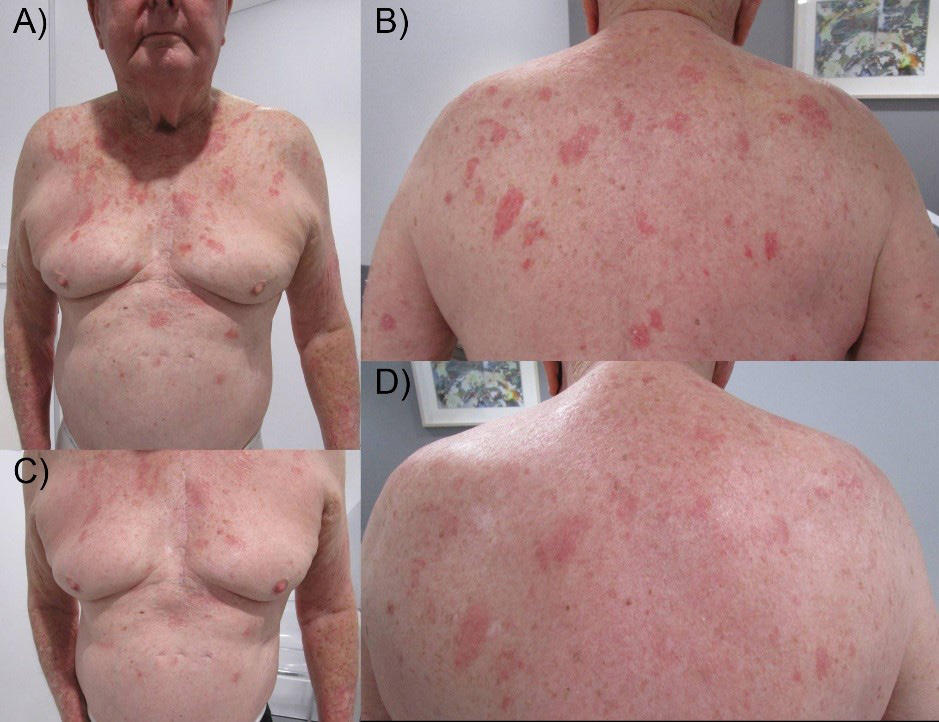
Severe plaque psoriasis of the body following dose 2 of the Oxford-AstraZeneca COVID-19 vaccine (A and B), improved with wet dressings over seven days (C and D).

AZ dose 2 was administered 84 days after dose 1. However, eleven days after dose 2, the most severe flare occurred with chest, upper abdomen, upper back, and face involvement (PASI 15.2) (Figure 1A and B), necessitating wet dressings in hospital for two days and at home for a further five days. The eruption then abated to PASI 2.8 (Figure 1C and D). Our patient was on uninterrupted tildrakizumab therapy throughout for over 6 months.

The close temporal link between AZ vaccination and onset of the psoriatic flares suggests a causal association between these events. The first and second psoriatic flares did not appear to be associated with AZ. Additionally, the second flare did not appear to be attributed to either influenza vaccine or AZ. However, the third and final flare occurred following completion of vaccination with AZ.

While the first flare may have been attributed to influenza vaccination, these have been reported as uncommon in a study by Munguía-Calzada et al.^4^ Furthermore, our patient’s influenza vaccinations over the past decade caused no issue.

Although not yet proven, severe allergic reactions and psoriatic flares may be attributed to polyethylene glycol (PEG) and its derivatives.^3,5^ While our patient’s COVID-19 and influenza vaccines did not contain PEG, a derivative, polysorbate 80, is a constituent of AZ, tildrakizumab, and influenza vaccines received by our patient. Polysorbate 80 has been associated with the development of severe non-immunological reactions and anaphylactoid reactions with cutaneous symptoms.^6^ However, this excipient is unlikely to have been the cause of flares as our patient has experienced no reaction to either previous influenza vaccines or ongoing tildrakizumab.

The possibility of the flare being a paradoxical eruption from tildrakizumab was discounted. To date, only urticarial eruptions have been reported,^7^ and not psoriasiform. Our patient experienced no further exacerbation as a result of treatment with tildrakizumab both before and after the reported flares.

The combination of AZ and pre-activation of natural killer (NK) cells from the influenza vaccine may have been contributory. One study demonstrated pre-activation of natural killer (NK) cells by inactivated H3N2 virus resulted in increased IFN-γ^+^ NK cells 30 days post-vaccination.^8^ NK cell-induced inflammation in psoriatic lesions may be based on keratinocyte activation via cytokines including IFN-γ and TNF-α.^9^ Although Australian influenza vaccines are reformulated annually,^10^ each formulation received by our patient contained this strain.

The underlying immunological mechanisms of systemic reactions to COVID-19 vaccines are still poorly understood. In the case of our patient, it appears AZ dose 1 may have predisposed to each exacerbation. Our report supports increasing evidence that psoriasis flares may result from COVID-19 vaccination even in well-managed disease. Therefore, closer monitoring may be indicated following each vaccination dose.

## Competing interests

None.

## Ethical approval

The patient in this manuscript has given written informed consent to publication of case details. This case study was conducted according to the ethical principles of the Declaration of Helsinki.

## Authors’ contributions

NT: Visualization, Writing- Original draft preparation, Editing.LJL: Conceptualization, Supervision, Writing- Reviewing and Editing.
